# Assessment of the retinal nerve fiber layer in individuals with obstructive sleep apnea

**DOI:** 10.1186/s12886-016-0216-2

**Published:** 2016-04-18

**Authors:** Blanca Ferrandez, Antonio Ferreras, Pilar Calvo, Beatriz Abadia, Jose M. Marin, Ana B. Pajarin

**Affiliations:** Department of Ophthalmology, IIS-Aragon, Miguel Servet University Hospital, Isabel la Catolica 1-3, 50009 Zaragoza, Spain; University of Zaragoza, Zaragoza, Spain; Department of Pneumology, Miguel Servet University Hospital, Zaragoza, Spain; Centro de Salud Seminario, Zaragoza, Spain

**Keywords:** Retinal nerve fiber layer, Obstructive sleep apnea, Optic nerve head, Optical coherence tomography, Scanning laser polarimetry

## Abstract

**Background:**

The effect of obstructive sleep apnea (OSA) syndrome in the peripapillary retinal nerve fiber layer (RNFL) thicknesses remains unclear. The purpose of this study was to assess RNFL measurements acquired using scanning laser polarimetry (SLP) and optical coherence tomography (OCT) in patients with OSA.

**Methods:**

The sample of this cross-sectional study included 40 OSA patients and 45 age-matched controls, consecutively and prospectively selected. All participants underwent at least one reliable standard automated perimetry (SAP) test, while RNFL measurements were obtained using the SLP and OCT. The OSA group was divided into 3 sub-groups based on the apnea/hypopnea index (AHI): mild, moderate, or severe OSA. SAP, SLP, and OCT outcomes were compared between the control and OSA groups. The relationship between AHI and RNFL parameters was also evaluated.

**Results:**

Age was not different between both groups. Mean deviation of SAP was −0.47 ± 0.9 dB and −1.43 ± 2.3 dB in the control and OSA groups, respectively (*p* = 0.01). RNFL thickness measured with OCT was similar between groups. OSA patients showed increased nerve fiber indicator (NFI; 20.9 ± 7.9 versus 16.42 ± 7.82; *p* = 0.01) and decreased superior average (59.74 ± 10.35 versus 63.73 ± 6.58; *p* = 0.03) obtained with SLP compared with healthy individuals. In the total sample, NFI and AHI were moderately correlated (*r* = 0.358; *p* = 0.001). In severe OSA subjects (*n* = 22), NFI and AHI had a Spearman correlation coefficient of 0.44 (*p* = 0.04).

**Conclusion:**

RNFL thickness measured with OCT did not differ significantly between groups. Severe OSA was related to a reduction of the RNFL thickness assessed by SLP.

## Background

Obstructive sleep apnea (OSA) syndrome is defined as a repetitive partial or complete obstruction of the upper airway during sleep [1]. OSA leads to hypoxia and hypercapnia [2], and is associated with cardiovascular, neurologic, and endocrine diseases [3]. Chronic hypoxia leads to an increase in hypoxia-inducible factor-1alpha and subsequent upregulation of a number of molecules, such as endothelin-1 and vascular endothelial growth factor, which stimulate neovascularization, weaken the blood–retina barrier, and induce local vasoconstriction of veins [4].

RNFL measurements provide a peripheral but accessible window to central nervous system neurons that could be damaged in association with OSA. Spectral-domain optical coherence tomography (OCT) provides quantitative and reproducible measurements for assessing RNFL thickness. Numerous studies have confirmed the ability of OCT to detect and monitor glaucoma [5–10], as well as to diagnose and follow-up other disorders that affect the optic nerve head (ONH), such as Parkinson disease, multiple sclerosis, and Alzheimer’s disease [11–14].

Scanning laser polarimetry (SLP) is a confocal scanning laser ophthalmoscope based on the principle that polarized light passing through the birefringent RNFL undergoes a measurable phase shift, known as retardation, which is linearly related to the RNFL tissue thickness [15]. The cornea also exhibits birefringent properties, which are significantly neutralized in SLP utilizing variable corneal compensation (VCC) [16]. Another confounder detected in RNFL birefringence measurements is the presence of atypical birefringence pattern images [17]. Thus, the latest generation of SLP, SLP with enhanced corneal compensation (ECC), includes an enhancement module that improves the performance of SLP-VCC for detecting RNFL damage [18–21] and progressive RNFL changes [22]. Recent studies revealed that the retardation measured by SLP is reduced prior to loss of RNFL thickness measured by OCT in experimental models [23–26].

Several studies have examined the relationship between OSA and RNFL thickness [27–35]. Most of these evaluated whether OSA patients have RNFL damage associated with chronic and intermittent hypoxia. Some reported reduced RNFL thickness in OSA patients based on OCT [28, 29, 31–33]. Ferrandez et al. [35] observed decreased retinal sensitivity based on white-on-white perimetry outcomes. Kargi et al. [27] and Moghimi et al. [30] reported decreased RNFL thickness in OSA patients measured with SLP compared with healthy subjects; both studies used older versions of SLP than we used in the present study (SLP-ECC).

To the best of our knowledge, this is the first study to evaluate and compare the RNFL loss measured by SLP-ECC and OCT in patients with OSA.

## Methods

The study protocol was approved by the Institutional Review Board (Clinical Research Ethics Committee of Aragon; CEICA), and informed written consent was obtained from all participants. The design of the study followed the tenets of the Declaration of Helsinki for biomedical research. The control group comprised individuals without abnormal ocular findings, obesity, or symptoms related to OSA. The OSA group was prospectively recruited from among patients at the respiratory department of our hospital.

The OSA group performed an overnight sleep study, which included electroencephalography, electromyography, electrocardiography, pulse oximetry measurements and respiratory movements. Sleep and respiratory events were recorded according to standard criteria [36]. OSA syndrome patients were defined as those with an apnea/hypopnea index (AHI) of at least 5 events/h. When the AHI was between 5 and 14 events/h was considered as mild OSA, when the AHI ranged from 15 to 19 events/h was considered as moderate OSA, and when the AHI was higher than 19 events/h was considered as severe OSA.

Inclusion criteria were best-corrected visual acuity of at least 20/40, refractive error within ±5.00 diopters equivalent sphere, and less than 2 diopters astigmatism; open anterior chamber angle; and transparent ocular media (lens opacity <1) based on the Lens Opacities Classification System III system [37]. Individuals with previous intraocular surgery, history of any ocular or neurologic disease, diabetes, previous treatment with non-invasive mechanical ventilation or oxygen therapy were excluded. Subjects with intraocular pressure higher than 20 mmHg, glaucomatous optic nerve head appearance, or glaucomatous visual field defects were also excluded.

### Study protocol

Participants underwent a full ophthalmologic examination: best-corrected visual acuity, slit-lamp biomicroscopy of the anterior segment, applanation tonometry (Goldmann), gonioscopy, ultrasound central corneal thickness measurement (OcuScan RxP; Alcon Laboratories Inc, Irvine, CA), and ophthalmoscopy of the posterior segment.

Two reliable standard automated perimetries (SAPs) were performed using a Humphrey Field analyzer, model 750i (Zeiss Humphrey Systems, Dublin CA). The 24-2 Swedish Interactive Threshold Algorithm (SITA) Standard program was selected. If fixation losses were greater than 20 % or false positive or false negative rates were greater than 15 %, the perimetry was repeated at least 3 days apart to avoid a fatigue effect. The SAPs were performed before any clinical examination or imaging test. The data from the second reliable SAP were used for the statistical analysis to minimize the learning effect.

The Cirrus OCT (Carl Zeiss Meditec, Dublin, CA; Optic Disc Cube 200x200; software version 6.2) was used to measure the peripapillary RNFL thickness after mydriasis (0.5 % tropicamide; Alcon Laboratories Inc, Fort Worth, TX). Images were focused and acquired when subjects were properly positioned. Only scans with a signal/strength ratio greater than 6/10 were accepted. Left eye data were converted to a right eye format.

The SLP-ECC (GDx PRO, Carl Zeiss Meditec, software version 1.0) was performed by the same operator following a standard protocol. All scans were acquired through undilated pupils with a low ambient light. Subjects were asked to keep their head still during each measurement, with their faces resting on the facemask to allow for the best alignment between the instrument’s anterior segment compensator and the eye position. A primary scan was obtained before each measurement to compensate for corneal birefringence.

The ECC algorithm introduced a predetermined large birefringence bias to shift the measurement of the total retardation to a higher value region to remove noise and avoid the problem of atypical patterns [38]. After image acquisition, the birefringent bias was removed mathematically, point-by-point, from the final RNFL image. Calculations were performed on a ring of fixed sized tissue centered on the ONH head automatically determined by the SLP-ECC software.

Images that were obtained during eye movement, unfocused and/or poorly centered images, and those with a quality scan score of less than seven were excluded. Good-quality images from SLP were defined by residual anterior segment retardation of 15 nm or less and an atypical scan score not greater than 25. SLP parameters used as outcome measures for this investigation included nerve fiber indicator (NFI), temporal-superior-nasal-inferior-temporal (TSNIT) average, superior average, inferior average, and TSNIT standard deviation (SD). The NFI is a global measure based on the entire RNFL thickness map, and it is calculated using a support vector machine algorithm based on several RNFL parameters. NFI ranges from 1 to 100, with lower values (around <25) indicating normal RNFL. Although some studies indicate that the NFI is the most sensitive parameter of SLP for glaucoma diagnosis [39, 40], its calculation method is based on various parameters and therefore it does not directly indicate RNFL thickness.

All tests were performed within 6 weeks of the participant’s date of enrollment into the study.

### Statistical analysis

Statistical analyses were calculated using the IBM SPSS (version 22.0; IBM Corporation, Somers, NY) statistical software. For all analyses, *p* < 0.05 was considered statistically significant. Only one eye per participant was randomly chosen, unless only one eye fulfilled the inclusion criteria. All study variables were normally distributed, as verified by the Kolmogorov-Smirnov test. Student’s t tests were used to compare demographics, SAP, OCT, and SLP-ECC parameters between groups. Pearson correlations were calculated between the AHI and OCT and SLP-ECC parameters, and Spearman correlations were calculated between severe OSA and OCT and SLP-ECC parameters.

## Results

The study sample comprised 45 controls and 40 OSA patients of Caucasian origin. Mean age was 48.67 ± 8.12 years in the control group and 46.30 ± 9.31 years in the OSA group (*p* = 0.21; Table 1). Intraocular pressure was significantly lower in the OSA group (17.42 ± 2.6 mmHg in healthy subjects and 14.23 ± 2.6 mmHg in OSA patients; *p* < 0.001) and best-corrected visual acuity was higher in the OSA group (0.93 ± 0.08 versus 0.98 ± 0.07; *p* = 0.006), although these differences were not clinically meaningful. Central corneal thickness did not differ between the groups (557.44 ± 33.61 μm in healthy subjects and 555.75 ± 23.44 μm in OSA patients; *p* = 0.79).

**Table 1:** Clinical characteristics of the study population

	Control		OSA		
	Mean	SD	Mean	SD	p*
Age (years)	48.67	8.12	46.30	9.31	0.214
BCVA (Snellen)	0.93	0.08	0.98	0.07	0.006
SE (Diopters)	−0.23	2.17	0.12	1.24	0.372
IOP (mmHg)	17.42	2.60	14.23	2.64	<0.001
Pachymetry (μm)	557.44	33.61	555.75	23.44	0.792
MD SAP (dB)	−0.47	0.90	−1.43	2.35	0.014
PSD SAP	1.43	0.21	1.83	0.76	0.001
VFI SAP	99.47	0.63	98.03	3.37	0.007
*n*	45		40		

*OSA* Obstructive sleep apnea, *SD* Standard deviation, *BCVA* Best-corrected visual acuity, *SE* Spherical equivalent, *IOP* Intraocular pressure, *MD* Mean deviation, *SAP* Standard automated perimetry, *PSD* Pattern standard deviation, *VFI* Visual field index, *n* number of cases

*Student’s *t* test

In the OSA group, the mean Epworth Sleepiness Scale score was 9.17 ± 4.31 (over 24). During the overnight sleep study, systolic and diastolic blood pressures were 121.34 ± 13.51 mmHg and 80.83 ± 8.08 mmHg, respectively, while minimum oxygen saturation was 80.63 ± 8.28 % (range: 54–94 %), and sleep latency was 0.27 ± 0.25 min (range: 0.03–1.30 min). The mean AHI was 41.46 ± 23.59 (range: 6–97).

Main indices of SAP were different between both groups. Mean deviation (−1.43 ± 2.35 dB versus −0.47 ± 0.9 dB; *p* = 0.01) and the visual field index (98.03 ± 3.37 versus 99.47 ± 0.63; *p* = 0.007) were lower in the OSA group, while pattern standard deviation was higher in the OSA group (1.83 ± 0.76 versus 1.43 ± 0.21; *p* = 0.001).

The OCT parameters revealed no differences between groups in peripapillary RNFL thickness at each of the 12 clock-hour positions, in the 4 quadrants, or in the average thickness (Table 2).

**Table 2:** Differences in peripapillary RNFL thickness measured by OCT between the control group and OSA group

	Control				OSA				
	Min	Max	Mean	SD	Min	Max	Mean	SD	p*
H1	58	161	117.15	21.96	30	163	112.72	24.18	0.394
H2	19	139	87.93	23.26	64	160	93.85	18.39	0.210
H3	15	83	58.43	13.51	45	77	59.90	7.82	0.552
H4	41	115	73.38	17.28	30	104	68.45	13.03	0.154
H5	61	162	108.55	25.69	81	210	120.03	29.37	0.067
H6	104	211	148.85	25.04	103	213	150.10	24.01	0.820
H7	11	180	141.35	28.22	92	181	134.93	21.99	0.260
H8	49	86	68.17	10.36	42	95	64.45	11.83	0.138
H9	34	73	50.15	8.21	36	66	51.78	6.85	0.340
H10	55	144	78.18	16.22	43	100	73.58	12.89	0.164
H11	102	184	132.28	18.50	10	169	122.37	30.21	0.081
H12	86	195	133.63	27.11	39	183	128.48	32.80	0.446
Superior	101	161	127.65	15.58	55	151	120.75	19.86	0.088
Inferior	101	165	133.70	16.10	110	171	134.13	15.61	0.905
Nasal	38	110	74.07	14.61	52	148	77.72	17.78	0.319
Temporal	47	88	65.32	8.95	51	79	63.95	7.55	0.460
Average thickness	84	120	100.20	8.53	61	114	97.00	10.43	0.137

Values are expressed in microns (μm)

*OSA* Obstructive sleep apnea, *Min* Minimum, *Max* Maximum, *SD* Standard deviation, *H* retinal nerve fiber layer thickness at every clock hour position for a right eye

*Student’s *t* test

Table 3 shows the comparison of SLP-ECC parameters between the healthy and OSA patients. NFI was higher in the OSA group (20.9 ± 7.9 versus 16.42 ± 7.82; *p* = 0.01), and the superior average was lower in the OSA group (59.74 ± 10.35 versus 63.73 ± 6.58; *p* = 0.03).

**Table 3:** SLP-ECC parameter values for control group and OSA patients. Values are expressed in polarimetric microns (p-μm), except for the NFI (learning classifier)

	Control group				OSA patients				
	Min	Max	Mean	SD	Min	Max	Mean	SD	p*
NFI	2	29	16.42	7.82	2	47	20.90	7.90	0.010
TSNIT Average	37.4	63.1	51.68	5.01	45.6	62.4	51.13	3.29	0.550
Superior Average	48.1	77.8	63.73	6.58	6.6	81.8	59.74	10.35	0.035
Inferior Average	45.9	87.4	64.70	7.73	53.2	78.2	63.40	6.36	0.402
TSNIT SD	17.3	34.2	25.52	3.75	14.8	35	24.25	3.97	0.132

*OSA* Obstructive sleep apnea, *Min* Minimum, *Max* Maximum, *SD* Standard deviation, *NFI* Never Fiber Index, *TSNIT* Temporal-Superior-Nasal-Inferior-Temporal

*Student’s *t* test

NFI obtained with SLP-ECC correlated moderately with the AHI with a Pearson correlation coefficient of 0.358 (*p* = 0.001). In the 22 severe OSA patients, NFI and AHI had a Spearman correlation coefficient of 0.44 (*p* = 0.04).

## Discussion

OSA syndrome has a reported prevalence of more than 20 % but the pathogenesis of neurodegeneration in OSA remains unclear [41, 42]. OSA is associated with arterial and pulmonary hypertension, neurovascular and cardiovascular disease, arrhythmia, and other systemic disorders [43].

OSA leads to secondary vascular dysregulation due to the hypoxia and hypercapnia produced during the apnea episodes. The hypoxia-hypercapnia process, the autonomic dysregulation, and the endocrine and hemodynamic changes lead to a state of oxidative stress, which is an important factor in many intermediary mechanisms of diverse pathologies. This dysregulation results in increased circulating blood endothelin-1 levels in the OHN among other tissues. Increases in circulating endothelin-1 are also associated with other diseases like multiple sclerosis [44], fibromyalgia [45], and transiently during optic neuritis [46].

Our hypothesis was based on the idea that ONH vascularization in OSA patients is compromised as suggested by the vascular theory proposed by Anderson [47], which states that axonal nutrition and axoplasmic flow are affected by impaired ONH microcirculation. Persistent or temporary ischemia can block the blood supply from the posterior ciliary arteries to the short posterior ciliary arteries, thus leading to non-perfusion of the anterior part of the ONH and neuronal ischemia. Continuous poor vascularization of the ONH during apneic episodes could alter the ONH morphology, which could be detected by OCT and SLP-ECC.

Previous studies, performed with different imaging devices, reported contradictory outcomes regarding the RNFL thickness in OSA patients [27–35]. Some of them found decreased RNFL thickness in OSA individuals [27–34], while others found no reduction [35, 48] or no relationship between the RNFL thickness and disease severity [30, 31].

Although we could not evidence changes in the RNFL thickness assessed by OCT, there were differences in the main indices of SAP, and NFI and superior average measured with SLP-ECC. The RNFL thickness at the superior quadrant measured by OCT did not reach statistical significance (*p* = 0.088), but tended to follow a similar pattern as the SLP-ECC findings (lower value in the OSA group), demonstrating that the results from the two methods are not at variance, but that SLP-ECC is more sensitive.

The RNFL thickness values acquired with these devices cannot be compared directly or used interchangeably, because the measurements are based on different optical techniques. The ability of SLP to detect RNFL defects has been widely validated [49–51], but some experimental studies suggest that the birefringence of the RNFL may be decreased prior to reduction of the RNFL thickness [23–26]. This characteristic of the RNFL would lead to better performance of the SLP-ECC compared with spectral-domain OCT to detect changes in OSA patients.

The OSA group exhibited a diffuse depression of the visual field, without typical patterns of other pathologies such as glaucoma [35]. It seems that OSA syndrome mainly leads to a dysfunction of the retinal ganglion cells instead of their death. Consequently, while the retinal anatomy is relatively preserved, SAP is more sensitive to detect the malfunction of the visual pathway than imaging test.

Huseyinoglu et al. reported worse mean indices of Octopus perimetry in OSA patients compared to healthy individuals [32]. Nevertheless, Lin et al. observed similar mean deviation of SAP (30–2 SITA Standard program) between the control and OSA groups [28]. Different protocol designs and samples make it difficult to compare the results among studies.

Recent evidence demonstrates that RNFL retardation measured by SLP is reduced earlier than RNFL thickness thinning measured by OCT in experimental models, including experimental glaucoma [17–20]. SLP assesses RNFL thickness around the optic disc. Because the technology is based on reflectivity, measurement is hampered by polarization of the ocular media, and this can lead to confounding by non-RNFL birefringence. Improvements in this technology, including ECC, have led to more reproducible results and more accurate discrimination between healthy and glaucomatous eyes [52, 53].

Although other authors have evaluated RNFL measurement using SLP-VCC [27, 30], to our knowledge, this is the first study evaluating RNFL thickness measured with SLP-ECC in OSA patients. Kargi et al. [27] used the SLP with a fixed corneal polarization compensator (FCC) and Moghimi et al. [30] used the SLP-VCC, whose measurements are not as accurate as those obtained using the SLP-ECC or spectral-domain OCT. Individual variations in corneal birefringence, which are not completely corrected by FCC, result in over- or underestimation of RNFL parameters [54]. Moghimi et al. [30] investigated the presence of ocular hypertension and glaucoma in OSA syndrome using functional and structural tests. They found that 6.7 % of OSA patients had an intraocular pressure higher than 21 mmHg, and 3.9 % of OSA patients had glaucoma, while the group included no patients with ocular hypertension or glaucoma. In our study, we excluded glaucoma patients or patients with intraocular hypertension because our purpose was to study the effect of intermittent hypoxia, which occurs in OSA syndrome, on the peripapillary RNFL thickness.

The NFI and superior average acquired with SLP-ECC differed significantly between healthy controls and OSA patients. It is noteworthy, however, that the NFI measurement is a machine learning classifier based on a linear support vector machine, not a parameter used to measure disease severity.

A limitation of our study was that healthy controls did not perform an overnight sleep study because this examination is costly. We assumed that the control group did not include individuals with apnea/hypopnea events, because obesity and symptoms related to OSA (fatigue, snoring, sleepiness, adenoid facies, etc.) were exclusion criteria for this group.

## Conclusions

In the present study, OCT did not demonstrate neurodegenerative changes in OSA patients, but SAP and some SLP-ECC parameters were significantly reduced in OSA patients compared with healthy individuals. Further prospective longitudinal studies are required to clarify the role of visual field, OCT and SLP-ECC as neurodegenerative markers in OSA syndrome.

## Ethics approval and consent to participate

The study protocol was approved by the Institutional Review Board (Clinical Research Ethics Committee of Aragon; CEICA), and informed written consent was obtained from all participants. The design of the study followed the tenets of the Declaration of Helsinki for biomedical research.

## Consent for publication

Not applicable.

## Availability of data and materials

Data will not be shared because the authors performed other analyses, which are not yet published.

## Abbreviations

AHI: apnea/hypopnea index
BCVA: best-corrected visual acuity
ECC: enhanced corneal compensation
FCC: fixed corneal polarization compensator
H: retinal nerve fiber layer thickness at every clock hour position for a right eye
IOP: intraocular pressure
MD: mean deviation
Max: maximum
Min: minimum
n: number of cases
NFI: nerve fiber indicator
OCT: optical coherence tomography
ONH: optic nerve head
OSA: obstructive sleep apnea
PSD: pattern standard deviation
RNFL: retinal nerve fiber layer
SAP: standard automated perimetry
SD: standard deviation
SE: spherical equivalent
SLP: scanning laser polarimetry
TSNIT: temporal-superior-nasal-inferior-temporal
VCC: variable corneal compensation
VFI: visual field index
